# Sustainable Nanotechnology Strategies for Modulating the Human Gut Microbiota

**DOI:** 10.3390/ijms26125433

**Published:** 2025-06-06

**Authors:** Gréta Törős, Gabriella Gulyás, Hassan El-Ramady, Walaa Alibrahem, Arjun Muthu, Prasad Gangakhedkar, Reina Atieh, József Prokisch

**Affiliations:** 1 Institute of Animal Science, Biotechnology and Nature Conservation, Faculty of Agricultural and Food Sciences and Environmental Management, University of Debrecen, Böszörményi Street 138, 4032 Debrecen, Hungary; hassanelramady@rocketmail.com (H.E.-R.); arjunvmuthu@gmail.com (A.M.); prasad25gkdkar@gmail.com (P.G.); joe.prokisch@gmail.com (J.P.); 2 Doctoral School of Animal Husbandry, Faculty of Agricultural and Food Sciences and Environmental Management, University of Debrecen, Böszörményi Street 138, 4032 Debrecen, Hungary; gulyas@agr.unideb.hu; 3 Soil and Water Department, Faculty of Agriculture, Kafrelsheikh University, Kafr El-Sheikh 33516, Egypt; 4 Doctoral School of Health Sciences, University of Debrecen, Egyetem tér 1, 4028 Debrecen, Hungary; walaaeb@mailbox.unideb.hu; 5 Institute of Agricultural Chemistry and Soil Science, Faculty of Agricultural and Food Sciences and Environmental Management, University of Debrecen, Böszörményi Street 138, 4032 Debrecen, Hungary; atieh.reina@mailbox.unideb.hu; 6 Department of Food Microbiology and Safety, College of Food Technology, Vasantrao Naik Marathwada Agricultural University, Parbhani 431402, India; 7 Doctoral School of Nutrition and Food Science, University of Debrecen, 4032 Debrecen, Hungary

**Keywords:** antimicrobial nanomaterials, prebiotic agents, byproducts, sustainable development

## Abstract

Antibiotic resistance remains a pressing global health concern, necessitating the development of sustainable and innovative antimicrobial strategies. Plant-based nanomaterials, particularly those synthesized from agricultural byproducts, such as mango seeds, tomato skins, and orange peels, have emerged as promising candidates due to their potent antimicrobial activity and reduced likelihood of resistance development. These nanomaterials exert their effects through diverse mechanisms, including the generation of reactive oxygen species, the disruption of microbial membranes, and interference with critical cellular functions, such as DNA replication. Beyond their antimicrobial properties, recent studies have demonstrated their ability to modulate gut microbiota composition—promoting beneficial genera such as, *Lactobacillus* and *Bifidobacterium*, while inhibiting pathogenic species like *Staphylococcus* spp. This dual functionality positions them as attractive agents for prebiotic interventions and targeted dietary strategies. The convergence of plant-derived nanotechnology and personalized nutrition, guided by individual microbiota profiles, offers a novel paradigm for enhancing host health and preventing infection-related disorders. This review provides a comprehensive overview of the sustainable production of nanomaterials from agricultural and food industry waste, their antimicrobial and prebiotic applications, and their potential in regulating gut microbiota. Furthermore, we discuss emerging nanoenabled strategies to combat infectious diseases and highlight future directions for mechanistic studies, safety assessments, and clinical translation in pharmaceutical, nutraceutical, and functional food contexts.

## 1. Introduction

Nanotechnology is an emerging interdisciplinary field with huge potential in health, agriculture, and environmental sustainability. A key trend is the utilization of agricultural and food industry byproducts as raw materials for synthesizing plant-based nanomaterials [1,2].

With diverse therapeutic potential, plant-based nanomaterials are emerging as critical functional ingredients in food and medicine. They exhibit antibacterial [3], antifungal [4], antihelmintic [5], and antiviral properties [6], making them valuable in combating antimicrobial resistance [7]. Additionally, their ability to stimulate probiotic growth underscores their role in gut microbiota modulation [8,9], which makes them valuable candidates in the fight against antimicrobial resistance (AMR) [10], a mounting global health threat primarily driven by the overuse of antibiotics and the resulting disruption of the gut microbiota [11,12].

Green synthesis of nanomaterials from agricultural waste offers a more sustainable and safer alternative to traditional chemical methods. While conventional synthesis often involves toxic solvents, high energy input, and hazardous byproducts, biosynthesis from agro-waste uses renewable resources and eco-friendly processes, reducing environmental costs and carbon footprints [13,14]. Agricultural residues, such as grape pomace [15], coffee grounds [16], and fruit peels [17], are rich in bioactive compounds (e.g., polyphenols, flavonoids, polysaccharides) that can act as reducing and capping agents, enabling the synthesis of stable, functional nanoparticles [18,19].

Nanotechnology is offering innovative solutions to some of the most pressing global challenges. In agriculture, its integration has transformed traditional practices, enabling sustainable solutions that address environmental, health, and economic concerns [2,20]. Among its promising applications is the ability to bridge the gap between agricultural byproducts and cutting-edge health interventions by using agro-derived nanomaterials to regulate gut microbiota [15,21] to face several waste management issues [1], like improper handling practices. Furthermore, the composition of its byproducts can also be affected during storage and transportation [22].

The human gut microbiota, a dynamic ecosystem of trillions of microorganisms, plays an essential role in digestion, immune regulation, nutrient metabolism, and even neurobehavioral functions. It is critical in digestion, nutrient absorption, immune modulation, and mental well-being [23]. Dysbiosis, or an imbalance in the gut microbiota, is associated with numerous health issues ranging from gastrointestinal disorders to metabolic syndromes and infectious diseases. As research advances, it has become clear that restoring and maintaining a balanced gut microbiota is essential for preventing and managing these conditions [24,25,26].

Our review explores the sustainable integration of nanotechnology and microbiota science, focusing on developing sustainable agro-applications of nanoparticles and producing nanomaterials from agro- and food industrial byproducts. It then delves into their applications in medicine and pharmacology, focusing on antimicrobial activity and prebiotic potential. The human gut microbiota composition and its connection to infectious diseases are also examined alongside innovative nanostrategies to develop disease resistance.

## 2. Methodology of the Review

To ensure a thorough and reproducible literature review, we systematically searched several academic databases, including ScienceDirect, SpringerLink, PubMed, and Google Scholar. Our search strategy used various keyword combinations like “nanomaterials”, “agricultural byproducts”, “prebiotics”, “prebiotic activity”, “antimicrobial activity”, and “gut microbiota”. We focused primarily on articles published between 2019 and 2024 to capture the latest developments in the field. We included only peer-reviewed original research articles and reviews written in English that explored the use of nanomaterials or nanotechnology derived from agricultural byproducts, particularly those related to gut microbiota regulation or the related biological effects. Studies were excluded if they were unavailable in full text, presented only as conference abstracts, or lacked sufficient methodological detail. We also considered factors, such as journal impact factor, author expertise, and relevance to our research question during the selection process. Articles were screened by title and abstract, then assessed in full for eligibility. The final selection of studies was organized and visually summarized using tables and figures to present key insights and trends.

## 3. The Agro-Journey of Nanotechnology

### 3.1. Sustainable Agro-Applications of Nanomaterials

The rapid growth of the global population has intensified pressure on agricultural systems to maximize production, leading to linear agrarian practices, globalized food supply chains, extensive storage networks, and large-scale agro-industrial processing. While these systems address food security, they generate vast agro-industrial and food waste [27]. Global food waste and losses are staggering, estimated at approximately USD 680 billion annually [28]. The Food and Agriculture Organization (FAO) reports that over 1.3 billion tons of food are discarded yearly, 25–35% of global food production [29]. Each year, more than 2 billion tons of agro-industrial waste—nearly 30% of global agricultural production—are generated from vegetable, fruit, dairy, and meat production, distribution, and commercialization chains [30]. Without proper management, these byproducts contribute to soil and water pollution, with long-term effects on human health and agroecosystems [31]. However, these materials also hold significant potential for bioenergy [32], biopolymers [33], bioplastics [34], biofertilizers [35], antibiotics [36], cosmetics [37], and food industry additives [38]. Integrating nanotechnology into agro-waste management offers a sustainable strategy for transforming waste into valuable resources [39,40].

The scale of agricultural waste generation varies globally. India produces approximately 620 million tons of agricultural waste annually, while China is the most significant global producer [41]. Common agricultural biomass waste includes rice straw, wheat straw, corn straw, sugarcane bagasse, and rice husk, with a respective global annual production of 731, 354, 204, 181, and 110 million tons [27]. The food industry further contributes significant byproducts, such as rapeseed meal (35 Mt), citrus waste (15.6 Mt), banana waste (9 Mt), grape pomace (5–9 Mt), and apple pomace (3–4.2 Mt) annually [42]. In South Africa, crop residues alone total 43 Mt per year, with maize contributing 28 Mt, followed by grain (32 Mt) and sugarcane (6 Mt). Oil crops (groundnut, sunflower, and soybean) generate around 3 Mt, while vegetables (tomato, potato, and cabbage) add approximately 1 Mt [29]. On a global scale, agricultural activities produce an estimated 140 Gt of biowaste annually, including agri-food and forestry residues [43].

Livestock production further compounds environmental concerns, contributing significantly to waste output [44]. Food waste alone accounts for approximately 3.3 billion tons of CO_2_ emissions annually—about 8% of human-induced greenhouse gas emissions [45]. Traditional disposal methods, such as landfilling, release harmful gases like methane and hydrogen sulfide. At the same time, incineration produces toxic byproducts, including dioxins and flue gases, that degrade air quality and public health [46]. The combined annual economic cost of food waste is estimated at USD 1 trillion, with environmental costs pushing this figure to USD 2.6 trillion [47].

Addressing these challenges demands innovative and sustainable solutions. Adopting zero-waste principles, recycling, and upcycling can mitigate environmental harm while promoting resource conservation and circular economy practices [48]. Efficient waste management strategies can significantly reduce carbon emissions, support climate change mitigation, and drive sustainability initiatives [49]. One promising approach involves nanotechnology. Nanomaterials offer unique capabilities in enhancing food production, improving waste management efficiency, and advancing sustainability efforts. For instance, metal nanoparticles can optimize enzymatic activity in composting and anaerobic digestion, accelerating the breakdown of complex organic compounds [20,50].

**Figure 1:**
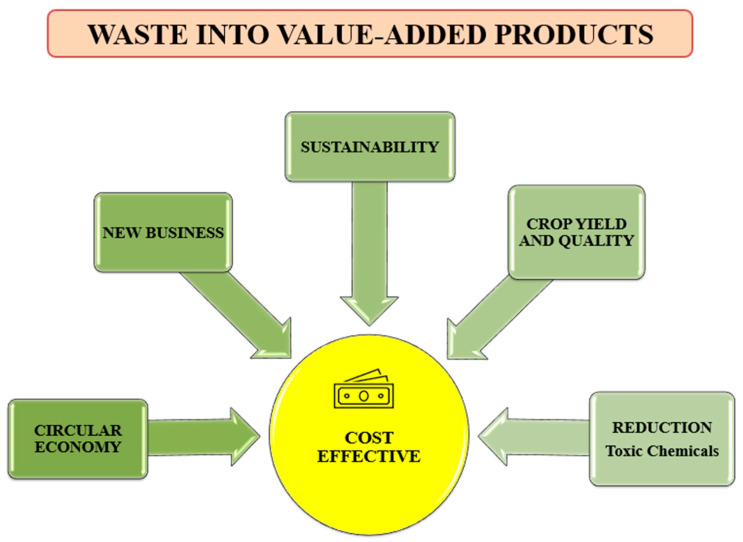
The economic benefits of plant byproduct-based nanomaterials. Sources: [52,53,54,55,56].

Valorizing agricultural and food waste through green synthesis presents a dual advantage: reducing environmental waste while generating high-value nanomaterials with applications across multiple industries [51]. This integration of advanced nanotechnologies into sustainable waste management frameworks has the potential to revolutionize global food systems, enhance food security, and mitigate environmental impacts. Plant-byproduct-based nanomaterials offer vast economic benefits, spanning cost savings in agricultural production, reduced ecological footprints, and emerging business opportunities. As illustrated in Figure 1, the increasing adoption of plant-based nanotechnology will be pivotal in driving new business opportunities, promoting sustainability and circular economy practices, improving crop yield and quality, and reducing the use of toxic chemicals. These advances will collectively build a more resilient and sustainable economic future while delivering significant environmental benefits.

### 3.2. Medicinal and Pharmacological Value of Sustainable Nanomaterials

Many researchers reported the relationship between agro-wastes and the production of nanomaterials [15,38,40,57]. Nanoparticles could be produced using bio-wastes, such as leaves, stems, seeds, pulp, and bagasse, as a green approach due to low energy consumption and low-cost production [30]. Therefore, the preparation of nanoparticles based on cellulose, pectin, metal (TiO_2_, Ag, ZnO, and others), or silica (i.e., organic, inorganic, or hybrid nano-composites) is presented in Table 1. In this table, the green production of different nanoparticles and their application can be seen. The green strategy of converting agricultural waste to other applications may include nanoenabled energy [58,59], cement-based construction materials [60], smart nanobiosensors [61], removing pollutants like antibiotics [62], biodegradable polymers [63], and antibacterial film production [64].

**Table 1:** Green production of nanoparticles using agro-wastes and their applications.

Nanoparticles (NPs)/Nanomaterials	Agro-Waste Kind	Suggested Applications	Ref.
Potassium-doped graphene oxide	Oak (*Quercus ilex*) fruit seeds	Antimicrobial activity	[65]
Graphene oxide-nano zero-valent iron	Sugarcane bagasse	Photo-catalytic removal of antibiotics in water	[62]
Al_2_O_3_ nanocatalyst	*Quercus incana* L. seeds	Feedstock for producing nanocatalysts and biodiesel	[66]
Gold nanoparticles (AuNPs)	Dried plum peel	Antibacterial activity against positive and Gram-negative bacteria	[67]
Wurtzite ZnO-nanoparticles	Sugarcane press mud	Photo-catalytic activity in the agricultural and environmental fields	[68]
Niobium oxide-NPs (Nb_2_O_5_-NPs)	Pecan nutshell, *Carya illinoinensis*	Antioxidant activity	[69]
Nanochitosan	Shrimp shell waste biomass	Antibacterial activity	[64]
Titanium dioxide-NPs (TiO_2_-NPs)	Banana pseudostem	Removal of Indigo Carmine dye	[70]
Nanomaterials of cellulose and lignin	Sorghum biomass	Accelerating the industrial and economic prospects of bio-based biorefineries	[71]
ZnO-NPs	Corn stalk pith	Bio-filters for the purification of water	[72]
Silver nanoparticles	Durian peel (*Durio zibethinus* Murr.)	Antibacterial activity against both negative- and positive-gram bacteria	[73]
ZnO-activated carbon nanoparticles	Plantain peel	Effective adsorbents for treatment of wastewater pollution	[74]
Calcium borate nanoparticles	Sugarcane bagasse	Enhancing seed germination and development	[75]

**Figure 2:**
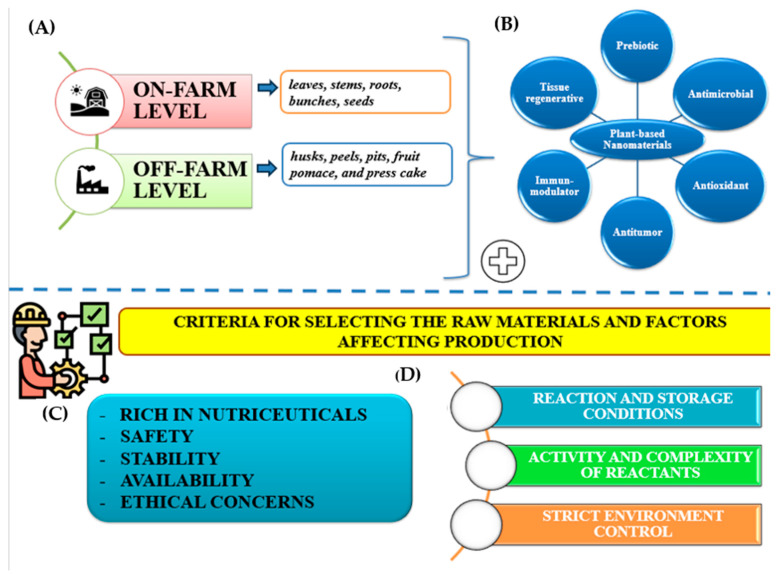
(**A**) The types of agricultural byproducts, (**B**) common health effects, (**C**) the most important criteria for selecting the raw materials, and (**D**) some significant factors affecting the production of plant-based nanomaterials. Source: [16,36,45,81,82].

Nanoparticle (NP) synthesis typically follows one of two main strategies: the “Top-Down” or “Bottom-Up” approach (as described at Figure 2A). The Top-Down method involves breaking down bulk materials into nanoscale structures through physical and chemical techniques, leading to size reduction. In contrast, the Bottom-Up approach (green synthesis) builds NPs from atomic or molecular precursors through processes (reduction and oxidation), resulting in nanoparticles with fewer structural defects and a more uniform chemical composition [76,77].

For instance, green synthesis and eco-friendly methods using natural materials, like neem leaves and banana peels, have shown potential in producing silver nanoparticles [78] with antibacterial and dye-degrading properties while reducing the impact of labor on the ecosystem by using environment-friendly solvents and reagents [50,78]. Nanoparticles also enhance the processing and utilization of phytochemicals in agro-industrial waste by improving their stability, dispersibility, bioavailability, and bioactivity [79].

These wastes fall into two main categories: agricultural residues (on-farm level) and industrial wastes (off-farm level), as seen in Figure 2A. Agricultural residues include field waste (leaves, stems, and seeds) and processing byproducts (husks and roots). Industrial food processing waste consists of materials, such as oils, peels, pomaces, and molasses [80], which offer several functional possibilities in human medicine (Figure 2B). Many researchers have described various health-related uses of nanoparticles (NPs) derived from agricultural byproducts. Several factors influence plant-based nanomaterials, including the nature of raw materials and some production parameters, like conditions and reactants, and environmental control, as presented in Figure 2C,D.

Agro-nanomaterials, synthesized from agricultural byproducts, represent a sustainable innovation in medicine and pharmacology. Their inherent biocompatibility, environmental abundance, and multifunctional properties position them as promising tools for addressing critical healthcare challenges [83,84]. These materials are successfully employed in drug delivery [85], bioimaging [86], wound healing [87,88], antimicrobial treatments [3], and as a biosensor to detect and monitor diseases [89,90].

Despite their numerous advantages, agro-nanomaterials face several challenges that must be addressed before their widespread adoption in medicine. For instance, some agro-nanomaterials degrade rapidly under environmental conditions, reducing their effectiveness [91]. While plant-based nanoparticles are generally considered safe, their interaction with human cells and tissues must be extensively studied to rule out potential adverse effects [92].

Recent studies underscore the pharmacological potential of nanomaterials derived from agricultural byproducts, demonstrating their efficacy as drug delivery systems while contributing to waste reduction and minimizing environmental impact. Their potential extends beyond pharmacology into biosensing [89], regenerative medicine [93], and antimicrobial treatments [94], offering a pathway toward more sustainable and effective therapeutic solutions.

Furthermore, many recent studies have been published on the synthesis process of agricultural byproducts from different points of view as follows: Apple pomace, a byproduct of Malus domestica, undergoes ultrasound-assisted chemical precipitation to yield starch nanoparticles, which exhibit antioxidant activity and gut microbiota regulation [95]. Coconut husk from *Cocos nucifera* is converted into nitrogen-doped carbon nanomaterial through hydrothermal-assisted thermal treatment, enhancing the sensitivity and specificity of electrochemical biosensors [96]. Grape pomace, derived from *Vitis vinifera,* is the source for cellulose nanocrystals, which are synthesized by isolating cellulose from a toluene/ethanol (2:1, *v*/*v*) extract and hydrolyzing it with 64% H_2_SO_4_.

This has potential in tissue engineering [93,97]. Oyster and Shiitake mushroom byproducts from *Lentinula edodes* and *Pleurotus ostreatus* undergo an acid-base procedure to form β-glucan nanoparticles, demonstrating antitumor potential against breast carcinoma and colon cells [98]. Rice husk, another *Oryza sativa* byproduct, is processed through delignification and acid hydrolysis treatment to obtain cellulose nanocrystals, exhibiting antioxidant, cellular antioxidant, and antiproliferative activities [99].

## 4. Antimicrobial Activity of Plant-Byproduct-Based Nanomaterials

### 4.1. Nanomaterials as Antimicrobial Agents Against Antibiotic Resistance

Antibiotic resistance has become a significant public health crisis, with only two new classes of antibiotics introduced in the past four decades. The emergence and spread of multidrug-resistant bacteria have intensified the problem, making infection control increasingly complex [100]. According to the World Health Organization [101], antimicrobial resistance is a “silent pandemic”, causing an estimated 700,000 deaths annually—a figure projected to rise to 10 million by 2050 if no significant interventions are made.

Plant byproduct-based nanomaterials have recently emerged as promising antimicrobial agents due to their multifaceted action mechanisms, making it less likely for bacteria to develop resistance. Studies have demonstrated the antimicrobial potential of various nanoscale materials, including liposomes, metal oxides, polymers, and antimicrobial peptides [102,103,104].

### 4.2. The Role of Plant Byproducts in Nanoparticle Synthesis

Increasing attention has been given to utilizing agricultural and food processing byproducts as raw materials for synthesizing nanoparticles with antimicrobial properties. For instance, Angamuthu [3] used mango seed waste (*Mangifera indica*) to biosynthesize silver nanoparticles via an ethanolic extract and silver nitrate solution, achieving notable antibacterial activity. Similarly, Hani et al. [105] synthesized AgNPs from aqueous extracts of orange peel (*Citrus sinensis*), which displayed strong antibacterial and antifungal activity, particularly against *S. aureus* and *E. coli*.

In most cases, plant extracts serve as reducing and stabilizing agents, capping the surface of nanoparticles with secondary metabolites (e.g., phenolics, terpenoids, flavonoids) that may enhance or complement the metallic core’s antimicrobial action [106], so a critical distinction must be made between the intrinsic antimicrobial effects of the metallic nanoparticle and the functionality of the bioactive phytochemicals derived from plant byproducts used during green synthesis.

### 4.3. Green Synthesis of Nanoparticles Using Plant Byproducts

The green synthesis of nanoparticles using agro-industrial residues and food processing byproducts are cost-effective and sustainable alternatives to chemical synthesis [73]. These plant materials contain an abundance of phytochemicals—including alkaloids, flavonoids, terpenoids, and phenolics—that facilitate metal ion reduction and nanoparticle stabilization [107], which in fact, can be supported by the following evidence:

- Rigopoulos et al. [108] employed olive mill waste (*Olea europaea*) as a reducing agent in silver nanoparticle synthesis through a factorial experimental design, reporting strong antibacterial efficacy against *E. coli* and *S. aureus*;
- Other notable examples include cellulose/ZnO nanoparticles derived from peanut shells (*Arachis hypogaea*), which showed enhanced antimicrobial effects, especially against yeast [109];
- Silver nanoparticles synthesized from tomato peel (*Solanum lycopersicum*) demonstrated considerable antibacterial activity [110].

Overall, these examples underscore the dual functionality of plant byproducts in nanomaterial fabrication: (1) enabling green synthesis and (2) potentially enhancing antimicrobial efficacy via bioactive surface moieties.

### 4.4. Mechanisms of Antimicrobial Action

**Figure 3:**
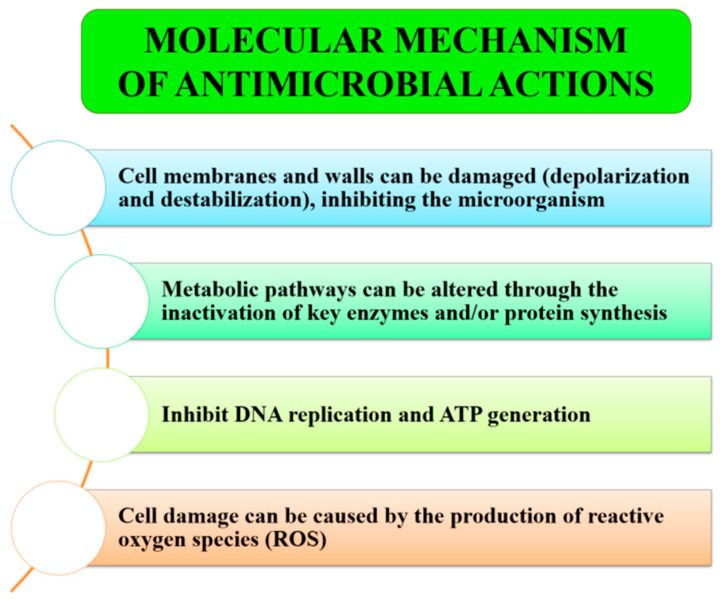
Suggested mechanism of the antimicrobial activity of nanoparticles. Source: [109].

Numerous studies have explored the mechanisms through which nanoparticles exert antimicrobial effects (Figure 3). These include the generation of reactive oxygen species (ROS), inhibition of ATP production and DNA replication, interference with protein synthesis, inactivation of key enzymes, and disruption of microbial membranes—ultimately leading to cellular destabilization and death [111]. Additional mechanisms involve interference with microbial metabolic pathways and the prevention of biofilm formation.

The antimicrobial activity of plant byproduct-based nanomaterials is generally attributable to two synergistic mechanisms: the physicochemical effects of the metallic nanoparticle core [112] and the biochemical contributions of phytochemical surface layers [113]. 

These plant-derived nanoparticles can generate ROS, release antimicrobial metal ions (e.g., Ag^+^, Cu^2+^, Zn^2+^), and inhibit microbial adhesion and motility—key steps in biofilm formation. ROS include highly reactive species, such as hydrogen peroxide (H_2_O_2_), hydroxyl radicals (•OH), and superoxide anions (O_2_•−). When microbial antioxidant defenses are overwhelmed, these species can damage critical cellular components, including proteins, lipids, RNA, and DNA [114,115,116]. The bacteriostatic effect of nanoparticles is often linked to releasing metal ions into the surrounding medium, disrupting enzymatic activities, impairing cellular respiration, and reducing microbial survival. Higher concentrations and prolonged exposure typically enhance these effects [117].

Nanoparticles also prevent early biofilm development by targeting microbial adhesion proteins and motility structures such as flagella. Furthermore, some metal-based nanoparticles inhibit enzymes involved in peptidoglycan synthesis, reduce microbial adhesion, and block biofilm maturation via sustained ion release [118].

Plant-based capping agents not only stabilize nanoparticles but can also enhance their antibacterial activity. These agents contain bioactive compounds such as polyphenols, saponins, or alkaloids with inherent antimicrobial properties [119]. Additionally, redox-active groups in phytochemicals can modulate ROS production [120] and improve nanoparticle dispersion and bioavailability in aqueous environments [121].

Beyond direct microbial inhibition, metallic nanoparticles, such as silver and zinc oxide, can influence the gut microbiota through complex host-mediated pathways. Once ingested, they may interact with intestinal epithelial cells by activating Toll-like receptors (TLRs), which trigger downstream signaling via the NF-κB pathway. This leads to the release of proinflammatory cytokines, such as TNF-α and IL-6, ultimately altering gut microbial composition by promoting or suppressing specific bacterial populations [122].

Moreover, nanoparticles may affect the production of microbial metabolites, including short-chain fatty acids (SCFAs) like acetate, propionate, and butyrate. These metabolites are essential for gut health, serving as colonocyte energy sources, enhancing epithelial barrier integrity, and modulating immune responses through G-protein-coupled receptors (e.g.: GPR43, GPR41) [45,122]. The interplay between nanoparticle-induced immune signaling and microbial metabolite production highlights the intricate relationship between nanomaterials and the gut microbiome.

### 4.5. Factors Affecting Antimicrobial Efficacy

The effectiveness of these nanomaterials largely depends on their physicochemical characteristics, such as surface area-to-volume ratio, size, shape, and metal density. Nanoparticles with smaller sizes generally exhibit more potent antimicrobial activity due to better penetration into microbial cells [123,124,125]. Several interdependent factors influence the overall antimicrobial performance of these hybrid nanomaterials:

Particle size and shape play an essential role. For instance, Shumbula et al. [126] reported that silver nanoparticles showed strong antimicrobial effects, particularly at smaller sizes, achieving minimum inhibitory concentrations as low as 3.5 μg/mL. Particle shape also plays a role; Alnehia et al. [127] found opening increased antimicrobial efficacy, possibly due to the induction of crystal defects. Oval-shaped Cu-TiO_2_ nanoparticles exhibited substantial activity against *S. aureus* and *E. coli* [128], while flower-shaped nanoparticles were more effective than rod-shaped ones [129].

In addition, the surface chemistry of nanoparticles can influence their effectiveness. Phytochemicals on their surface introduce functional groups, such as hydroxyl and carboxyl, that can interact with microbial membranes or proteins, potentially enhancing antimicrobial action [130].

Synthesis variables—including solvent type, pH, temperature, and precursor concentration—can significantly influence the antimicrobial properties of the resulting nanoparticles. These properties include producing ROS, disrupting membranes, inhibiting enzymatic functions, and blocking access to essential micronutrients. Some metals can also directly interact with microbial DNA [131,132,133,134,135].

### 4.6. Comparative Summary of Antimicrobial Activity

Table 2 represents the dual contribution model described above. The antimicrobial efficacy observed in many systems likely results from the core metal and the plant-derived phytochemicals.

### 4.7. Challenges and Opportunities

Despite their promise, nanoparticle systems face several challenges: Although nanoparticles can be synthesized through physical and chemical routes, these approaches often require significant energy inputs and utilize toxic reagents such as organic solvents and non-biodegradable stabilizers. These factors raise safety and environmental concerns, particularly when nanoparticles are intended for biomedical use, compared with green synthesis using plant byproducts [142].

Plant-based synthesis offers a sustainable alternative, leveraging phytochemicals such as alkaloids, terpenoids, flavonoids, and other functional groups (e.g., hydroxyl, carboxylic, sulfhydryl) that act as both reducing and stabilizing agents during nanoparticle formation [143]. However, the exact role of plant-derived compounds in nanoparticle stability, bioactivity, and interaction with biological systems remains under-investigated. Understanding the effects of metallic ions and plant bioactives is essential for rational design [144]. Furthermore, developing standardized synthesis protocols is a key step toward clinical or industrial application [145].

From a toxicological perspective, several essential questions about green-synthesized nanoparticles remain unanswered. One primary concern is how long these nanoparticles stay in the intestinal tract, which could affect local toxicity and their potential to enter the bloodstream [146]. Nanoparticles can interact with gut microbes or intestinal cells, which might lead to inflammation or weaken the gut barrier [147].

There is also worry about their ability to cross biological barriers like the intestinal lining, the blood–brain barrier, or even the placenta. Furthermore, depending on their size, surface charge, and coatings, nanoparticles could travel through the body and accumulate in organs, such as the liver, spleen, or brain [148], concerning their long-term safety and compatibility with the body [149].

These gaps make it difficult to gain regulatory approval and public trust. To move forward safely, we need more detailed in vivo and in vitro studies on how nanoparticles move through the digestive system, where they go in the body, how cells take them up, and how they are eventually eliminated. Advanced imaging tools and realistic biological models could help fill these gaps.

## 5. Exploring the Prebiotic Potential of Plant-Based Nanomaterials

### 5.1. The Role of Prebiotics

Prebiotics are indigestible food components that stimulate the growth of beneficial bacteria (e.g., bifidobacteria, lactic acid bacteria) in the gastrointestinal tract. They mainly consist of dietary fibers and oligosaccharides. They play a role in maintaining gut health and are known for their anticancer, immune-boosting, and cholesterol-lowering effects while reducing the risk of obesity [150,151].

**Figure 4:**
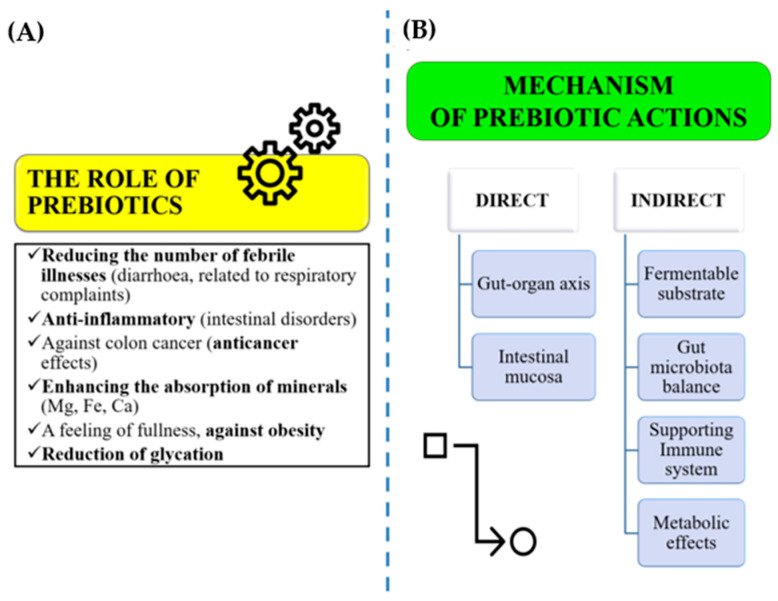
(**A**) Some agricultural byproducts are potential sources of prebiotics, and (**B**) their role in human health. Source: [150,152].

Figure 4A illustrates the role of plant-based prebiotics in promoting human health. Prebiotics influence host health via two main mechanisms: indirect and direct effects. Indirectly, prebiotics act as fermentable substrates for beneficial gut bacteria, helping to maintain a balanced gut microbiota. This microbial activity, in turn, positively impacts metabolic functions and modulates the immune system. Directly, prebiotics interact with the intestinal mucosa, modulating host cell signaling pathways and strengthening the integrity of the epithelial barrier. Furthermore, the gut communicates with various organs—including the brain, heart, lungs, liver, pancreas, bones, skin, muscles, reproductive system, kidneys, and bladder—as depicted in Figure 4B [152,153].

### 5.2. Plant-Based Byproduct Nanomaterials as a Strong Prebiotic

Several agricultural and food industrial byproducts are increasingly recognized as valuable sources for producing plant-based nanomaterials with significant prebiotic potential. Numerous studies have explored plant-derived prebiotic substances, with several promising results in stimulating probiotic growth, which can also be found in vast amounts of these byproducts. These substances can act synergistically [154] and exhibit more substantial prebiotic effects than commercially available prebiotics, such as inulin [155], fructooligosaccharides [156], and pectin [73]. These synergistic effects are attributed to gut microbes, like *Lactiplantibacillus plantarum* subsp. *plantarum* fermentability, and physicochemical properties, such as their nanoscale size, enable closer interaction with bacterial membranes and host epithelial cells [157].

For instance, nanocellulose derived from wheat straw and rice husk has demonstrated the ability to increase *Lactobacillus* populations in vitro significantly [158]. At the same time, pectin nanoparticles from citrus peel waste have enhanced short-chain fatty acid (SCFA) production and epithelial barrier function in intestinal cell models [159]. Lignin nanoparticles from sugarcane bagasse and corn bran, enriched with polyphenolic compounds, have been shown to support selective microbial growth and reduce oxidative stress in gut environments [160]. Moreover, these nanomaterials often provide additional functionalities beyond prebiotic activity. They can serve as delivery carriers for encapsulated probiotics or bioactive compounds, offering protection against stomach acid and targeted release in the intestine [161]. This dual functionality—acting as both prebiotic substrate and protective carrier—makes plant-based nanomaterials highly attractive for synbiotic formulations.

## 6. Exploring Preclinical and Early Clinical Evidence for Plant-Derived Nanoparticles

While most existing studies on plant byproduct-based nanomaterials remain in vitro or utilize animal models, an emerging body of research is beginning to explore their applicability in preclinical and early-phase human contexts, particularly for antimicrobial therapies. These plant-derived nanoparticles (PDNPs) combine the bioactivity of medicinal plants with the enhanced delivery and stability that nanotechnology provides.

Table 3 summarizes selected evidence from the literature, highlighting preclinical and early clinical studies evaluating the antimicrobial effectiveness of PDNPs. The materials are synthesized using extracts from *Azadirachta indica* (neem), *Camellia sinensis* (green tea), *Punica granatum* (pomegranate). These findings demonstrate outstanding potential for translational medicine, especially in wound healing, oral health, and oncology applications where microbial infection plays a key role in disease progression and treatment resistance [160,161,162,163,164,165].

## 7. Composition of Human Gut Microbiota and Infectious Diseases

### 7.1. Gut Microbiota and Its Role in Health

Recent explorations into the therapeutic potential of gut microbiota have shed light on mechanisms of colonization resistance, where a healthy microbiome prevents infections by outcompeting pathogenic bacteria for resources or directly inhibiting their growth [168]. Particularly notable is the implementation of non-systemic antibiotics that target localized infections while minimizing disruption to the gut flora. This approach is critical in controlling the emergence of antimicrobial resistance, a growing concern in clinical settings [169]. Moreover, leveraging gut microbes for their protective feature against infections offers a fresh perspective on infection management strategies, particularly in critically ill patients, where the risk of multidrug-resistant organisms (MDROs) is heightened by antibiotic pressure [170]. Therefore, ongoing research into gut microbiota profiles and their functional roles could illuminate new infection prevention and treatment pathways, moving beyond conventional antibiotics alone [171]. Pursuing gut microbiome-targeted therapies represents a paradigm shift toward personalized medicine in infectious and inflammatory disease management [172].

Recent classifications have referred to the gut microbiota as a “vital organ” because of its intricate, bidirectional interactions with other organs through neural, endocrine, humoral, immunological, and metabolic pathways. Changes in the microbial composition can cause gastrointestinal issues and affect diseases in various organs. However, the mechanisms behind the gut microbiota’s interactions with other organs are not fully understood [23,173].

### 7.2. Composition and Diversity of Gut Microbiota

The human gut microbiota is a diverse community of microorganisms that plays a key role in digestion, metabolism, immune response, neural signaling, and overall health [174,175]. Its composition exhibits considerable variability among individuals but generally includes several key components. The predominant constituents of the healthy gut microbiota are bacteria belonging to various phyla, with up to 90% of the phyla being *Firmicutes* and *Bacteroidetes* [176].

The phylum *Firmicutes* encompasses multiple genera, with the predominant genera, including *Lactobacillus, Bacillus, Enterococcus, Ruminicoccus,* and *Clostridium*, accounting for approximately 95% of its representation [177]. Bacteroidetes are composed of genera like *Bacteroidetes* and *Prevotella* [178]. Conversely, the genus *Bifidobacterium* represents the most prevalent group within the *Actinobacteria* phylum; however, the overall occurrence of this phylum is relatively low [179].

In addition to the bacteria, archaea are less abundant (1.2% of the microbial community in the gastrointestinal tract). Still, methanogenic archaea, including species, such as *Methanobrevibacter*, are notably present, particularly in the colonic regions of specific individuals [180]. Among the fungi, yeasts and molds, especially those in the genus *Candida*, are also part of the gut microbiota, although their particular roles remain less clearly defined [181]. Viruses (bacteriophages) in the human gut infect bacterial cells and possess the potential to influence both bacterial community dynamics and metabolic functions [182]. Unicellular eukaryotes like Entamoeba may also inhabit the gastrointestinal tract [183].

Various factors influence the gut microbiota composition, including diet, age, genetic predisposition, environmental conditions, lifestyle, and antibiotics [184]. A diverse microbiota is generally associated with favorable health outcomes, while dysbiosis—characterized by microbial imbalance—has been linked to various conditions, including obesity, Crohn’s disease, ulcerative colitis, and diabetes [185,186].

### 7.3. Gut Microbiota and Infectious Diseases

The human gut microbiota is an integral part of the immune system and overall health, with its composition significantly influencing susceptibility to infectious diseases. Infectious diseases, including malaria (caused by *Plasmodium*, is a genus of unicellular eukaryotes), AIDS (caused by human immunodeficiency virus), viral hepatitis (hepatitis B and C virus), tuberculosis (the pathogen is *Mycobacterium tuberculosis*), gastrointestinal infections (such as *Serovar Typhimurium* and *Clostridium difficile*), and coronavirus disease caused by SARS-CoV-2 pose a significant threat to public health [187]. Contemporary anti-infective therapies frequently do not succeed in achieving complete eradication of pathogens and may disrupt the equilibrium of the host microbiome. Such disruptions can contribute to a rise in the prevalence of drug-resistant pathogens, hasten evolutionary dynamics among these organisms, and potentially aggravate the severity of infectious diseases. Consequently, it is imperative to elucidate the roles and mechanisms of gut microbiota in the development, progression, and prediction of infectious diseases, and to evolve innovative strategies for preventing and treating these diseases in the future. The connection between gut microbiota and contagious diseases is mediated through several key factors. Notably, gut microbiota interacts with the immune system by promoting the production of diverse immune cell types (regulatory T cells, B cells, macrophages) and different signaling molecules (cytokines) [188]. A complex and diverse microbiota is associated with enhanced immune responses, offering improved defense against pathogenic organisms. A healthy gut microbiota can inhibit pathogens through competitive exclusion, competing for essential resources and attachment sites within the gastrointestinal tract, diminishing the likelihood of infection [189]. Additionally, gut microbiota contribute to homeostasis by producing short-chain fatty acids and diverse metabolites that bolster gut barrier function and modulate inflammatory responses. These metabolites may also regulate immune responses, potentially mitigating excessive inflammation that could precipitate disease [190].

Dysbiosis, characterized by an imbalance in gut microbiota composition, can compromise the integrity of the gut barrier and the immune system’s functionality, rendering the host more vulnerable to infections. For instance, dysbiosis has been linked to an elevated risk of gastrointestinal pathogens and systemic infections resulting from bacterial translocation [190].

Recent research indicates that gut microbiota may influence vaccine efficacy by modulating immune responses. A well-balanced microbiota has been shown to enhance antibody responses to vaccinations, thereby improving protection against infectious agents [191]. Furthermore, disruptions in gut microbiota due to antibiotic use may facilitate the proliferation of antibiotic-resistant pathogens, complicating infection management [192], while a balanced gut microbiota is crucial for immune function and disease protection. Probiotics, diet, and lifestyle changes can support microbiota health and boost defenses [187].

## 8. Nanostrategies to Develop Disease Resistance

Integrating nanotechnology into agricultural and food sciences has opened novel avenues for improving human health, particularly through gut microbiota modulation. Recent research highlights the potential of utilizing agro and food industry byproducts to create functional nanomaterials to enhance disease resistance. These nanointerventions present a sustainable, circular approach to transforming waste into high-value therapeutic tools—to promote human health. Table 4 provides an overview of the diverse applications of nanotechnology in promoting gut health and enhancing disease resistance, highlighting key innovations in the field.

It is worth mentioning that mushrooms can be an alternative tool to fight against antimicrobial resistance. In the future, it will be essential to explore the interactions between plant- and fungus-derived nanoparticles and the human microbiome, particularly through advanced metabolomic and functional microbiome analyses. Our research group has previously investigated several aspects of carbon nanodots derived from *Pleurotus ostreatus* (oyster mushroom), which provides a strong foundation for these future directions. For instance, we have successfully synthesized carbon-rich nanomaterials through pyrolysis, which exhibited potential for antimicrobial applications, with unique morphological features and appear well-suited for targeted microbial interactions in biomedical settings [210].

Based on our findings, future studies should examine the metabolomic effects of plant- and fungal-derived nanoparticles on the gut microbiome. Developing in vitro fermentation models will also be essential for better understanding this complex biological matrix.

## 9. Discussion and Conclusions

The growing challenge of antibiotic resistance has driven the search for alternative antimicrobial strategies, with plant-derived nanomaterials emerging as a promising solution. Sourced from agricultural byproducts, such as mango seeds, tomato skins, and orange peels, these nanomaterials exhibit strong antimicrobial properties without contributing to resistance development. Their mechanisms of action, such as generating reactive oxygen species and disrupting microbial membranes, enable them to combat a broad spectrum of pathogens effectively.

**Figure 5:**
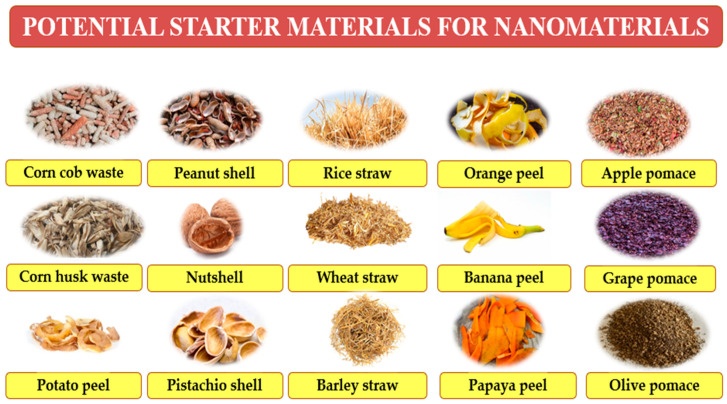
Overview of some potential agricultural byproducts recommended for nanomaterial synthesis and further investigations on gut microbiota (in vivo and in vitro).

As previously discussed, byproducts derived from plants offer a sustainable and valuable resource for the production of nanomaterials. Examples of such applications are illustrated in Figure 5. In addition to their well-known antimicrobial properties, these plant-based nanomaterials have shown promising capabilities in influencing the composition and function of the gut microbiota. By selectively promoting beneficial bacteria like *Lactobacillus* and *Bifidobacteria* while inhibiting harmful microbes, they may serve as innovative prebiotic agents to restore microbial balance. This dual function aligns with the growing interest in the diet–host–gut microbiota axis and its impact on overall health and disease prevention.

As research advances, incorporating these nanomaterials into personalized nutrition strategies tailored to individual microbiota profiles may offer more effective and sustainable health interventions. Nevertheless, comprehensive studies on their mechanisms of action, safety, and clinical relevance are essential to unlock their potential in functional foods and therapeutic applications fully.

In summary, plant-derived nanomaterials from agricultural byproducts represent a valuable opportunity for developing next-generation prebiotic and synbiotic formulations, offering enhanced efficacy, stability, and broad health benefits.

Further research is needed to clarify these nanomaterials’ long-term safety profiles, biodegradability, and potential host–microbe interactions. Additionally, interdisciplinary studies combining nanotechnology, microbiology, and nutrition science will be essential for translating laboratory findings into real-world applications. The green synthesis of pure carbon nanodots from various agricultural wastes should be prioritized to address environmental concerns and promote sustainability.

Standardizing production methods and investigating dose-dependent effects across diverse populations remain significant challenges. Addressing these open questions will help integrate plant-derived nanomaterials into food systems and clinical practice.

## Figures and Tables

**Table 2:** Plant-based NPs synthesized using different plant extracts and their antimicrobial activity.

Size Range	Type of NPs	Plant Species	Precursors Used	Shape & Size	Inhibition Against	Method	Ref.
<15 nm	Silver-Copper NPs	*Aerva lanata*	AgNO_3_ & CuSO_4_	Semi-spherical cluster, avg. 9.5 nm	*S. aureus*, *P. aeruginosa*	Agar well diffusion	[117]
	Silver-Tin Oxide NPs	*Zingiber officinale*	SnCl_4_ & AgNO_3_	Cubic crystal, avg. 9.5 nm	*S. aureus*, *E. coli*, *F. oxysporum*, *F. graminearum*	Disc diffusion	[136]
15–30 nm	Copper Oxide NPs	*Zizyphus spina-christi*	CuSO_4_	Spherical, 13.4–30.9 nm	*F. solani*	Agar dilution	[137]
	Silver NPs	*Vigna mungo*	AgNO_3_	Cubic, avg. 24.49 nm	*E. coli*, *S. aureus*	Agar well diffusion	[138]
	Silver NPs	*Hibiscus sabdariffa*	AgNO_3_	Spherical, avg. 21.22 ± 5.17 nm	*E. coli*, *S. aureus*	Disc diffusion	[139]
	Copper Oxide NPs	*Aloe barbadensis*	Cu(NO_3_)2·3H_2_O	Shape unspecified, <20 nm	*L. monocytogenes*, *K. pneumoniae*, *S. typhi*, *P. aeruginosa*	Agar well diffusion	[140]
>30 nm	Silver NPs	*Peganum harmala*	AgNO_3_	Oval, 42–72 nm	*S. typhi*, *P. aeruginosa*, *E. coli*, *B. subtilis*, *S. aureus*, *C. albicans*, *A. niger*, *P. notatum*	Micro dilution	[141]
	Gold NPs	*Peganum harmala*	HauCl_4_	Spherical, 12.6–35.7 nm	*S. typhi*, *P. aeruginosa*, *E. coli*, *B. subtilis*, *S. aureus*, *C. albicans*, *A. niger*, *P. notatum*	Micro dilution	[141]

**Table 3:** Preclinical and early clinical evidence for the antimicrobial effectiveness of plant-derived nanoparticles.

Extract Source	Nanoparticle Type	Application	Study Phase	Key Findings	Ref.
Neem Leaf	Silver (AgNPs)	Wound healing in diabetic ulcers	Pilot Clinical	Reduced wound size and microbial load	[162]
Pomegranate Peel	Silver (AgNPs)	Oral antimicrobial rinse	Early Clinical	Biofilm reduction in healthy volunteers	[163]
Green Tea	Gold/AgNPs	Oral biocompatibility, antimicrobial	Phase I	No adverse effects; biofilm inhibition	[164]
Neem+ Green Tea	Various NPs	General antimicrobial, wound care	Preclinical	Biocompatibility, microbial inhibition	[165]
Neem+ Pomegranate)	Silver NPs	Cancer models	Preclinical	Anticancer activity, ROS-mediated apoptosis	[166]
Green Tea (Catechins)	Polymer NPs	Oncology–breast/prostate cancer	Early Clinical	Enhanced efficacy, fewer side effects	[167]

**Table 4:** Applications of nanotechnology in agriculture and gut health.

Application Field	Description	Ref.
Nanotechnology in Agriculture	- Enhancing disease resistance and promoting human health by transforming waste into valuable therapeutic tools.	[30]
Nanoencapsulation Technologies	- Enhances stability, bioavailability, and delivery of prebiotics, probiotics, and synbiotics. Byproducts, such as cellulose and metal oxides, provide antimicrobial properties that benefit food preservation.	[30]
Gut Microbiota and Disease Resistance	- Target pathogenic microbes while preserving beneficial gut bacteria, such as Bifidobacteria and Lactobacilli. This helps maintain a balanced microbiome and supports gut health.	[193,194]
Prebiotic Nanoencapsulation	- Nanoencapsulation of prebiotics, such as inulin and fructooligosaccharides, enhances their stability and bioavailability, improving their effectiveness in the digestive system.	[195,196]
Probiotic Nanoencapsulation	- Probiotics can be protected using nanotechnology, such as alginate-based coatings and bionanocomposites, which increase their effectiveness by protecting them during gastric transit and enhancing their lifespan.	[197,198]
Synbiotic Nanoencapsulation	- Synbiotics (a combination of prebiotics and probiotics) are encapsulated using nanotechnology to improve their stability and therapeutic effects. They can potentially restore microbial balance and prevent infections.	[193,199]
Functional Foods and Bioactive Components	- Functional foods enriched with nanoparticles, such as polyphenols and carotenoids, improve gut health by enhancing bioavailability and promoting balance in the gut microbiome.	[200,201]
Nanoenhanced Functional Foods	- These foods boost the production of short-chain fatty acids (SCFAs) and other beneficial metabolites. They help lower inflammation, improve gut barrier function, and contribute to disease resistance.	[202,203]
Nanoagricultural Developments	- Innovations in nanoagriculture alter the gut environment by promoting beneficial bacteria growth, suppressing pathogens, and enhancing immune responses, leading to better disease resistance.	[204,205]
Bioactive Chemicals Synergy	- Nanoparticles combined with bioactive chemicals like polyphenols and prebiotics synergize to reduce oxidative stress, inflammation, and dysbiosis, helping to prevent and treat gastrointestinal illnesses.	[206,207]
Innovations in Probiotic Delivery	- Techniques such as alginate-based coatings, bionanocomposites, and pullulan nanoparticles derived from agricultural waste improve probiotics’ stability and antimicrobial properties.	[208,209]
